# Creating exclusive breastfeeding knowledge translation tools with First Nations mothers in Northwest Territories, Canada

**DOI:** 10.3402/ijch.v75.32989

**Published:** 2016-12-09

**Authors:** Pertice Moffitt, Raissa Dickinson

**Affiliations:** 1 Health Research Programs, Aurora Research Institute, Aurora College, Yellowknife, NWT, Canada; 2 Student Practicum, Aurora College, Yellowknife, NWT, Canada

**Keywords:** breastfeeding, rates of breastfeeding, remote settings in Canada, health promotion, knowledge translation

## Abstract

**Background:**

Breastfeeding is an ideal method of infant feeding affecting lifelong health, and yet the uptake of breastfeeding in some Indigenous communities in Canada's north is low.

**Objective:**

The aims of this project were to determine the rate and determinants of exclusive breastfeeding in a remote community in the Northwest Territories and to create knowledge translation tools to enhance breastfeeding locally.

**Methods:**

The study methodology followed three steps. Firstly, a series of retrospective chart audits were conducted from hospital birth records of Tłı̨chǫ women (n=198) who gave birth during the period of 1 January 2010 to 31 December 2012. A second follow-up chart audit determined the rate of exclusive breastfeeding and was conducted in the local Community Health Centre. Chart audit data included the following factors related to breastfeeding: age of mother, parity, birthweight and Apgar scores. Secondly, semi-structured interviews with a purposive sample of Tłı̨chǫ mothers (n=8) and one Elder were conducted to identify breastfeeding practices, beliefs and the most appropriate medium to use to deliver health messages in Tłı̨chǫ. Third, based on the information obtained in Step 2, two knowledge translation tools were developed in collaboration with a local community Advisory Committee.

**Results:**

The rate of exclusive breastfeeding initiation in the Tłı̨chǫ region is less than 30%. Physiological and demographic factors related to breastfeeding were identified. Thematic analysis revealed two overarching themes from the data, namely, “the pull to formula” (lifestyle preferences, drug and alcohol use, supplementation practices and limited role models) and “the pull to breast feeding” (traditional feeding method, spiritual practice and increased bonding with infant).

**Conclusion:**

There are a myriad of influences on breastfeeding for women living in remote locations. Ultimately, society informs the choice of infant feeding for the new mother, since mothers’ feeding choices are based on contextual realities and circumstances in their lives that are out of their control. As health care providers, it is imperative that we recognize the realities of women's lives and the overlapping social determinants of health that may limit a mother's ability or choice to breastfeed. Further health promotion efforts, grounded in community-based research and a social determinants framework, are needed to improve prenatal and postnatal care of Indigenous women and children in Canada.

Indigenous children in Canada have disproportionately elevated rates of morbidity including gastroenteritis, otitis media, respiratory infections and childhood-onset Type II diabetes compared to non-Indigenous Canadians (1–4). Addressing Indigenous health disparities by engaging people locally, actively and inclusively to help improve health outcomes is important (5). The promotion of breastfeeding, especially exclusive breastfeeding,^1^ has been shown to be the most important infant and child survival intervention in terms of its potential impact on morbidity and mortality (6–9). While the outcome of successful breastfeeding includes improved health and well-being for both the mother and the child, the alternative of feeding formula is associated with both short- and long-term health risks for the mother and the child (10). Despite the benefits of breastfeeding, there are numerous factors that influence the uptake, exclusivity and duration. Some of these factors include age of the mother and infant, the demanding nature of breastfeeding, lack of education, insufficient milk supply, painful breasts, employment or school interference, and personal and family attitudes (11).

In 2013, community service providers and researchers worked together to identify maternal health services, specifically breastfeeding resources for Tłı̨chǫ^2^ mothers in the Northwest Territories, Canada. The Aurora Research Institute and the Tłı̨chǫ Community Services Agency jointly applied for the placement of a Student Public Health Officer to facilitate a maternal knowledge translation project as a result of this cooperation. The project was also propelled by the simultaneous commitment from the Government of the Northwest Territories to early childhood development as well as interest from the territorial hospital that was at the time hoping to achieve Baby Friendly^3^ breastfeeding designation. The purpose of this paper is to share the process and results of the project developed by the Aurora Research Institute and Tłı̨chǫ community service providers targeting the promotion of breastfeeding with local Dene women in one region of the Northwest Territories.

## Methods and methodology

### Research aim

The aims of this project were to determine the rate and determinants of exclusive breastfeeding in a remote region in the Northwest Territories and to create knowledge translation tools to enhance breastfeeding locally.

### Study design

The study included three steps: a series of retrospective chart audits, semi-structured interviews with a purposive sample of Tłı̨chǫ mothers and one Elder, and the development of two knowledge translation tools.

### Retrospective chart audit

Firstly, we conducted retrospective chart audits with the goal to determine the rate of exclusive breastfeeding among Tłı̨chǫ mothers and to identify some factors that may affect breastfeeding (number of term births (12), birthweight (13,14), mother's age (15), Apgar^4^ scores and the type of delivery (17)).

A paper chart audit tool (Appendix A) was created, which recorded characteristics of the cohort of mothers, factors that may affect breastfeeding and if the baby was exclusively breastfed after birth or if supplementation was given. To test our data extraction tool, a pilot chart audit was conducted in February 2013 at the Mary Adele Bishop Health Centre in Behchokǫ̀ with permission of the health authority. A formal chart audit was conducted at Stanton Territorial Hospital in Yellowknife, Northwest Territories, with the birth records of Tłı̨chǫ women who gave birth during the period of 1 January 2010 to 31 December 2012 (n=198). The NWT Bureau of Statistics recorded 74 Tłı̨chǫ births in 2010 and 76 births in 2011 (18). If we estimate that this statistic remained constant, and the mean of these two numbers M=75 is used, then 88% of Tłı̨chǫ births were captured in this audit. The charts were pulled by Medical Records staff, and the audit was conducted by the researchers in a room on site in Medical Records, Stanton Territorial Hospital.

The tool had some limitations that were based on the accuracy of documentation on the chart. For example, the acronym G.T.P.A.L (gravida, term, preterm, abortion and living) was commonly used on the patient chart and provided salient data, but on six charts this information was missing. Also documentation did not capture the mother's intent to breastfeed. This question offered limited results and therefore was discarded. Three charts did not have documentation on Apgar scoring since the mother delivered at the Community Health Centre and was transported to the hospital following delivery. This information was missing from the chart.

A third chart audit was also performed again at the Mary Adele Bishop Health Centre in April 2013 using the charts for Well Baby visits, which occur from 2 weeks to 1 month after birth. Due to the loss of follow-up (mothers do not always choose to attend the Well Baby Clinic) and access to babies in one community only, the number of charts for the Well Baby visits was lower (n=67). Charts were audited on site in the largest Community Health Centre. Charts were pulled by the Centre staff.

Statistical descriptive analysis was conducted using Statistical Package for the Social Sciences (SPSS). Using this software, standard deviation, mean and maximum and minimum scores were calculated for Apgar, age, birthweight, term deliveries, para and gravida on the hospital record data. The frequency of feeding was also calculated for exclusive breastfeeding, exclusive formula feeding or breast and formula feeding.

#### Semi-structured interviews

Eight interviews were conducted in person with Tłı̨chǫ mothers aged between 20 and 40 years who were feeding an infant under 1 year of age. All participants gave birth during the time of the study; mothers varied in their infant-feeding practices, sometimes even between their own children in formula or breastfeeding. One Elder was interviewed to gain a historical perspective of breastfeeding in the Tłı̨chǫ region. Purposive sampling was used to select participants, and recruitment was facilitated by prenatal staff at the health centre and the Canadian Prenatal Nutrition Coordinator. Each participant was asked to describe her breastfeeding practices, beliefs and the most appropriate medium to use to deliver health messages in Tłı̨chǫ. Data analysis was conducted through a process of reading the transcripts several times in their entirety, line by line coding, creating categories and then themes to translate the findings into a usable format (19).

#### Knowledge translation tools

A Community Advisory Committee, consisting of one prenatal nurse, community health representatives, the same Elder participant and the health services director, advised the development of the knowledge translation materials. The role of the committee was to identify both the type of knowledge translation mediums to use (i.e. video, print) and perspectives on what elements would make educational materials culturally appropriate to the Tłı̨chǫ community.

**Table 1:** Tłı̨chǫ mothers January 2010 to December 2012 (n=198)

Number of term births	Total number	%
0	42	21
1–2	82	41
3–4	37	19
5–6	26	13
7–8	4	2
> 9	1	0.5
Not captured	6	3
Mother's age		
19 & younger	43	22
20–30	72	36
31–40	61	31
41 & older	3	1.5
Not captured	19	
Birthweight (mean=3,444 g)		
1,000–2,000 g	4	4
2,100–3,000 g	37	18
3,100–4,000 g	125	63
> 4,000 g	32	15
Apgar scores		
1 minute 8–10	162	82
1 minute 6–7	19	10
1 minute 4–5	6	3
Score 3 or less^a^	9	5

a Most of the nine babies in the 1 minute range of 3 and less recovered to 9 or 10, but one baby had scores of 0,1,1 and was on intravenous and tube feedings. Two other babies had scores of 2,5,6 and 3,4,5.

### Ethical considerations

Ethics approval was obtained from Stanton Territorial Ethics Committee and Aurora College. A research license from Aurora Research Institute was also received.

## Results

Statistical analysis of the chart audit at Stanton Territorial Hospital identified the following characteristics of Tłı̨chǫ mothers: 21% were first-time mothers (see Table 1); 41% experienced one to two full-term births to date while 34.5% of the mothers had more than three full-term births with 13% having five to six full-term births. One mother had 12 full-term births. Of the mothers, 22% were teenagers (19 years and younger). The mean birthweight was 3,444 grams with a range of 1,375–4,870 g. Large for gestational age (LGA) babies greater than 4,000 g represented 15% of babies born, while 5.6% were small for gestational age (SGA) less than 2,500 g. Birthweight is one of the several considerations of the health status of the newborn in terms of the babies’ abilities to feed via bottle or breast. Furthermore, the Apgar scores of the newborns are another consideration in terms of health status that would affect their ability to breastfeed. Of the babies in this cohort, 82% were found to have Apgar scores of 8–10 at 1 minute, and the mean was 8.6 with a standard deviation of 1.1. Finally, 88% of the babies were spontaneous vaginal deliveries, 8% were delivered by Caesarean section and 4% of babies were forceps or vacuum-assisted deliveries.

Of the Tłı̨chǫ mothers (n=198) who gave birth between 1 January 2010 and 31 December 2012, 60.1% initiated breastfeeding while 39.9% did not. Mothers initiating breastfeeding were 60.1%, but only 26.8% breastfed exclusively in the hospital while 33.3% supplemented feedings with formula. Narrative nurses’ notes revealed that nurses were using the words “topped up” to describe their intervention of supplementing breastfed babies with an additional 15–30 mL of formula without explanation or indication of rationale.

The Well Baby audit of 67 charts within the same cohort suggested that 35.5% of babies were breastfed while 64.5% of babies were only fed formula by the time of their first infant check-up. Of infants who were breastfed, 18.6% were also supplemented by formula. Eight charts audited were excluded from the sample because of missing data.

### Pull to formula: results from semi-structured interviews

#### Lifestyle preferences

Tłı̨chǫ mothers reported breastfeeding as an obstacle to returning to school or work after childbirth. In many cases, the pregnancy was unplanned and was an interruption to their schooling and current way of life. One participant stated “my friends do not breastfeed because they have no time. He never took from the bottle so I couldn't go back to school.” The message from her peers was that breastfeeding is demanding and time consuming.

#### Drug and alcohol use

Substance use and abuse was identified as negatively influencing the decision to breastfeed. A participant told us “people have bad habits, like addictions that stop them from breastfeeding, alcohol, smoking, chewing tobacco, and pot are very common habits.” Although the chief and councillors have declared alcohol prohibited, the prevalence of substance abuse is common. Some participants confirmed that mothers are making the best decision for their babies to bottle-feed so that the babies are not exposed to substances.

#### Supplementing breastfeeding

In the hospital, Tłı̨chǫ mothers were both formula and breastfeeding. Babies would feed from the breast and then be “topped-up” with formula. Women supplemented breastfeeding with formula for two reasons: to make sure that the baby was “full” or to expedite the hospital discharge process if breastfeeding was taking too long to learn. Some participants never initiated breastfeeding or stopped due to difficulties with producing milk, latching or physical pain. As one participant expressed, “I did not breastfeed, I tried for two weeks but the baby would not latch and I was too sore so I had to quit.” Inadequate milk supply and the experience of my “milk would not produce but my boobs were so full” was noted by multiple women.

#### Advertising formula

Some Tłı̨chǫ mothers believed that formula was better than breast milk. One participant referred to selecting a brand of formula “A+ is the best formula. I think it is made with real breast milk.” The message on the can states “as good as breast milk.” Clearly, this messaging has an impact on some community mothers.

#### Breastfeeding support

Other participants fed their children formula because they did not have peer or family support to breastfeed. One participant mentioned that she grew up while residential schools were still open and that “grandma's kids were already big and there were no kids around in the community so I never saw breastfeeding.” While babies are still celebrated and beloved in communities, the ceremonies and the practice of intergenerational knowledge transmission have not continued as they once did prior to colonialism and, in particular, the residential school system which separated grandmothers, mothers and children and negatively impacted local customs and knowledge transmission of these customs.

### Pull to breastfeeding: results from semi-structured interviews

#### Traditional practice

Participants identified breastfeeding as both a spiritual and a traditional way of feeding babies. Some mothers were encouraged to breastfeed because it is traditional, “my family never said nothing, though they were happy I was breastfeeding it is how our ancestors would do it in the past.” Support for breastfeeding was also identified to be a spiritual process where “family did not help me breastfeed, God helped me. How I feel, God helped me.” Traditionally, babies were celebrated when they were born and welcomed with ceremony into the community.

#### Economical option

Some mothers breastfed because it was their only option. As one participant described, “I had no money and didn't work so I had to breastfeed I had no choice.” The price of formula is a burden for mothers who are unemployed and on social assistance.

#### Increased bonding with infant

Mothers told us breastfeeding nurtures a special bond with baby, and as an Elder stated, “the mother knows and belongs to the baby and the baby knows and belongs to the mother.” While breastfeeding, Elders encourage the new mother to talk and sing to the baby, and then the baby hears her mother's Tłı̨chǫ language and voice, which, the Elder believes, strengthens a child's identity. Mothers also suggested that breastfeeding helps babies control how they would like to be fed, as one participant explained, “I breastfed my four children and let each of them decide when it was time to stop. In total, I breastfed till my children were 7 to 8 months old.”

#### Healthy practice

Even if they did not breastfeed their children themselves, most participants identified breastfeeding as a healthy practice. The benefits of breastfeeding are shared at local prenatal classes at the Community Health Centre. The women we interviewed have received the message that “breast is best.”

### Knowledge translation tools

Unanimously, the committee agreed that visual materials, storytelling, and seeing and doing are the traditional ways of learning and were the best way to translate breastfeeding information to the Tłı̨chǫ community. We proceeded to develop an informational breastfeeding photobook and video (see Appendix B).

In the photobook, we used pictures of the community and Tłı̨chǫ women breastfeeding. The photobook layout and video script were created by the researchers and emanated from a Western academic lens. This became apparent with a review by the committee. For example, we had put a photograph on the cover of the book, a dome-like structure in the community that sits on the shores of Marion Lake. We learned that the structure was jokingly called “the nipple” by the community and was deemed as an inappropriate picture. Instead, they recommended an art piece of mother and infant from a local artist. We also had a photograph of bison, which we thought was relevant in terms of the meaning of the land and animals to local people. We were wrong. An Elder reminded us that in their language the word “for bison” translates “to mean cow,” and local people suggest that if you drink formula you will become “wild like the cow,” a very different message than what we were trying to convey.

The committee also routinely reviewed the video to ensure culturally appropriateness. The video was filmed with the help of a group of local Tłı̨chǫ researchers known as the Community Action Research Team (CART). CART has utilized arts-based materials in many projects reporting their effectiveness in community capacity-building (18). We hired actors from Behchokǫ̀ to appear in the film and posted the film on the Tłı̨chǫ community website. The video was shared locally with mothers attending the Behchokǫ̀ community prenatal classes and also with nurses at the Community Health Centre

In both materials, we addressed the determinants of breastfeeding that were identified during interviews with participants and have been previously discussed in the pull to formula and pull to breastfeeding results sections. Negative breastfeeding myths, such as formula containing real breast milk, were also dispelled. Special attention was given in both tools to highlight the cultural nuances and importance of breastfeeding in the community.

### Limitations

The retrospective chart audit provides a cross-sectional picture of only one group of mothers in the Northwest Territories (NWT) and by no means is a generalization of patterns and practices of breastfeeding in the entire territory. The entire project was focused on and aligned with a student practicum experience. It was time sensitive. Mothers from only one of the four Tłı̨chǫ communities participated in the interviews.

## Discussion

The results of the chart audit point to a low uptake of exclusive breastfeeding. This is similar to the results of the Canadian Maternity Experiences Survey, which found a national exclusive rate of breastfeeding of 14.4% at 6 months (20). Suboptimal exclusive breastfeeding is also noted in other regions of the Circumpolar North (21–24). Results from the Nunavut Inuit Health Survey reveal that the majority of Inuit babies receive suboptimal exclusive breastfeeding (25). Also a study conducted with Norwegian–Somali and Norwegian–Iraqi infants in Norway reported low exclusive breastfeeding at 4 months (23).

Encouragingly, a high mean Apgar score from the cohort of 8.6 is an indication of the health of babies born in the region. Low Apgar scores correlate with neurological compromise and negatively impact an infant's ability to breastfeed (26). Moreover, this finding suggests that babies in the cohort are healthy enough to initiate breastfeeding after birth.

Young mothers interviewed described competing agendas operating in their lives in terms of their own identities and stages of life. For example, custom adoption^5^ influences the uptake of breastfeeding with Tłı̨chǫ mothers since babies adopted to family or community members are formula fed. Similar influences of adoption on patterns of breastfeeding were identified in the northern region of Greenland (24) and in Nunavut, Canada (25).

Furthermore, the intersection of social determinants of health for Tłı̨chǫ women in the areas of income, employment, housing, education, health care services, substance abuse and violence is gravely affecting their well-being (28). Loppie and Wein suggest that disparities are experienced in unique ways and social determinants can be viewed through proximal,^6^ intermediate^7^ and distal^8^ levels (24). All these three levels are critical in the Tłı̨chǫ communities creating a context of increased suffering, burden of chronic disease, disempowerment and decreased capacity for personal decision-making. All Tłı̨chǫ mothers we interviewed were aware of the health benefits of breastfeeding but were juggling socio-economic pressures such as the desire to return to school and substance use.

Some research suggests that breastfeeding has a protective effect against the development of diabetes (30), particularly for First Nations children (31). It is important to note the rising rates of obesity and diabetes in First Nations communities (32), and the related scarcity of affordable healthy food choices and frequency of low-cost high-calorie market foods (33). A case in point is a study in Quebec, Canada, where anxiety in Cree mothers to food security was associated with anaemia, smoking and bottle-feeding (34). Furthermore, we identified that most babies are born with a healthy weight in terms of Canadian standards but there are a significant number of babies who are either SGA (5.5%) or LGA (15%), pointing to the link to obesity and diabetes that have been identified as a concern and call for action within other First Nation communities (32,35). The promotion of breastfeeding can help combat this epidemic (36).

Women who initiated breastfeeding told us they switched to formula when they noted pain or insufficient milk production. Inadequate milk supply has been noted as one of the most common reasons for early weaning^9^ in our study as well as others (37) and is the result of poor lactation management (38). Women did not feel supported with these challenges, and there were no lactation consultants and few breastfeeding coaches to help them. This gap was confirmed by the chart audit.

In our data, we found that women initiated formula feeding in the hospital for two main reasons. Firstly, women told us that formula was promoted with the “top-up” practice of the nurses. In hospital, exclusive breastfeeding education has been implemented. Secondly, women take up formula feeding in the hospital because they might be able to learn it quicker than breastfeeding and return to their communities sooner. Underlying this point is the medical travel policy for pregnant women in the Northwest Territories. The policy categorizes all pregnant women in the North as high risk and thus requiring hospitalization at 36–37 weeks, which in effect establishes childbirth within a medical model with inherent risks rather than a traditional and normal practice that benefits from family and community support (39).

Finally, as a result of this project, enhancements to maternal care for Tłı̨chǫ women have been recognized and in some cases initiated in the community. These activities include a peer support breastfeeding group and in-home prenatal outreach by a local nurse. While the usefulness of the tools (photobook and video) has not yet been formally evaluated, the establishment of the breastfeeding support group and the initiation of the home prenatal outreach are positive actions. An Elder also suggested that the bilingual photobook should be read and shared with youth to educate, strengthen and revitalize language.

## Conclusion

Numerous societal, community and personal factors influence a Tłı̨chǫ mother's decision to breastfeed. All of these factors were considered and included in the development of community-specific knowledge translation tools. The promotion of maternal health, specifically breastfeeding in addition to consistent work to improve socio-economic and living conditions, poverty reduction strategies and efforts to increase educational attainments are undeniably important (2,40) and have great potential to improve health. As noted by local people, the future of a Nation is rooted in the health of its children, and exclusive breastfeeding is a means of reaching this goal.

## Footnotes

1 Exclusive breastfeeding is described by the World Health Organization as feeding whereby the infant receives only breast milk for the first 6 months of life. After 6 months, solid food may be introduced but mothers are encouraged to continue to breastfeed up to 2 years and beyond.

2 Tłı̨chǫ are First Nations people who fall within the broader designation of Dene, Indigenous people of the widespread Athapaskan language family. Their name for themselves is *Doné*, meaning “the People.” Retrieved from http://www.thecanadianencyclopedia.ca/en/article/tlicho-dogrib/ The Tłı̨chǫ are Dene people who traditionally inhabited the lands from the borders of Nunavut in the east, the shores of Great Slave Lake in the north, the southern shores of Great Bear Lake and west towards the Mackenzie River in Canada's Northwest Territories. They are a self-governing nation with a population that includes the communities of Behchokǫ̀, Whatì, Gamètì and Wekweètì and is approximately 3,000 people (12). The Tłı̨chǫ nation is over 39,000 km^2^, the largest block of land owned by First Nations in Canada.

3 Baby-Friendly Hospital Initiative (BFHI) is a movement that was started in 1991 by the World Health Organization to promote and support breastfeeding in institutions around the world by implementation of the best tools, education and processes. Retrieved from http://www.who.int/nutrition/topics/bfhi/en/ The BFHI was initiated in the NWT in 2014 after this research was conducted and reported to the GNWT breastfeeding network. Both the photobook and the video were presented to members of the breastfeeding network along with an oral presentation of the results. It should be noted that the BFHI was pursued for many years in the NWT before coming to fruition.

4 The Apgar score is estimated at 1 and 5 minutes following birth to evaluate the physiologic status of the newborn. The score is derived from five parameters including heart rate, respiratory effort, response to irritation stimulus and colour (16).

5 Custom adoption has occurred for generations as a means of fortifying kinship ties, accommodating lifestyle requirements, keeping children in the community, gifting of a baby between relatives, and perhaps even more significant is the openness and contact that continues between the biological parents and the adopted parents. Custom adoption continues today and since 1995, a local Adoption Commissioner appointed through the Aboriginal Custom Adoption Recognition Act accepts applications from local people so that a certificate can be issued. This is needed so legal documents can be obtained, such as health care cards (27).

6 Proximal determinants of health “include conditions that have a direct impact on physical, emotional, mental or spiritual health” (28, p. 5). Examples were provided, such as overcrowding and family violence. In terms of breastfeeding, these barriers of overcrowding and family violence take an emotional toll on mothers attempting to feed their infants without the quiet space or support they require. Also there is a need to overcome sociopolitical barriers such as the easy access, promotion and marketing of infant formula. The Lancet Breastfeeding Group (29) has published a series of papers that relate to all three levels of determinants.

7 Intermediate determinants are those that create the proximal determinants, such as poor infrastructure and limited resources (28, p. 15). The cost of formula places an added burden on mothers who already have limited funds.

8 Distal determinants are those that construct the proximal and intermediate levels, such as “colonialism, racism and social exclusion” (28, p. 20). The colonial system has created the need for NWT mothers to travel for birth out of their communities to regional centres which causes isolation from families and interferes with supports that they need from their families and their community for breastfeeding. Also mothers do not have access to lactation consultants who could assist them with interventions to address physical discomforts associated with breastfeeding.

9 Weaning is defined as a process of a young infant to take food from a source other than a mother's breast.

## Appendix A

Chart audit template

1. Mother's parity
2. Birth weight
3. Mother's year of birth
  ○ 1940–60 ○ 1961–1980 ○ 1981–2000 ○ 2001–
4. Apgar score
5. Type of delivery
  ○ Spontaneous ○ Forceps/vacuum ○ Caesarean section
6. Was this mother intending to breastfeed on admission?
  ○ yes ○ no ○ undecided
7. Was breastfeeding initiated? ○ yes ○ no
8. Was supplementation given?
    a) If yes, what was given? (check all appropriate responses)

Formula

## Appendix B

Cover of photobook
